# Ewing Sarcoma of the Vagina: A Rare Clinical Entity

**DOI:** 10.7759/cureus.56550

**Published:** 2024-03-20

**Authors:** Arup Ganguly, Vaidarshi Abbagoni, Shravan Narmala

**Affiliations:** 1 Internal Medicine, University of Connecticut School of Medicine, Farmington, USA; 2 Internal Medicine, St. Vincent's Medical Center, Bridgeport, USA; 3 Hematology and Oncology, Doctors Hospital at Renaissance Health, Edinburg, USA

**Keywords:** extraskeletal, gynae oncology, ewing sarcoma family of tumors (esft), chemotherapy failure, sarcoma soft tissue

## Abstract

Ewing sarcoma (EwS), a malignancy primarily affecting adolescents and young adults, encompasses various types such as bone, extraskeletal, chest wall, and soft tissue-based tumors, all of which share a common genetic origin. A small portion of them are extraosseous, impacting diverse anatomical sites. Characterized by a specific translocation, this rare cancer rarely involves the vagina, with very few documented cases. This report details the unique case of a middle-aged woman diagnosed with extraosseous vaginal EwS, a rarity in this age group and gender. With no established guidelines, a multidisciplinary approach is crucial, emphasizing the need for further case reporting to enhance understanding and management strategies.

## Introduction

Ewing sarcoma (EwS) is a primary tumor of the bone that occurs primarily in adolescents aged 15 to 19 and young adults. On rare occasions, they may occur extraosseously with the involvement of other soft tissue sites like the lung, lower extremities, paravertebral spaces, head and neck, and the chest wall [1]. A review of the literature shows that there are fewer than 40 known cases of this tumor, and only a few are confirmed by molecular studies [2-4]. Over 90% of all EwS exhibit the t(11;22)(q24;q12) translocation, which fuses the EwS gene on chromosome 22 with the FLI1 gene on chromosome 11. 5 to 10% of EwS exhibit EWSR1-ERG translocation [t(21;22) (q22;q12)], while the others are less common [5,6]. There is not much data on the molecular analysis of tumors originating in the vagina owing to the rarity of the disease.

## Case presentation

**Figure 1:**
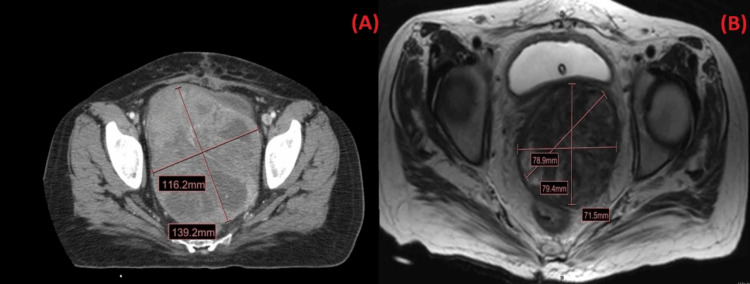
The CT scan and MRI of the pelvis and abdomen A: Heterogeneous mass measuring 11.6x13.9x11 cm in the pelvic floor without gross invasion of the surrounding structures and no associated lymphadenopathy; B: MRI of the abdomen and pelvis shows a notable shrinkage in the size of the mass to 7.9x7.9x7.1 cm

A 53-year-old postmenopausal woman with a past medical history of uterine fibroids post-hysterectomy presented to the emergency room with a history of vaginal bleeding and pelvic pain for four months. A pelvic exam performed was significant for a solid, irregular, and vascular mass extending from 1 cm proximal to the introitus to the vaginal apex. A biopsy of the mass was taken and sent to the pathology lab. A CT scan of the abdomen and pelvis is shown in Figure 1.

**Figure 2:**
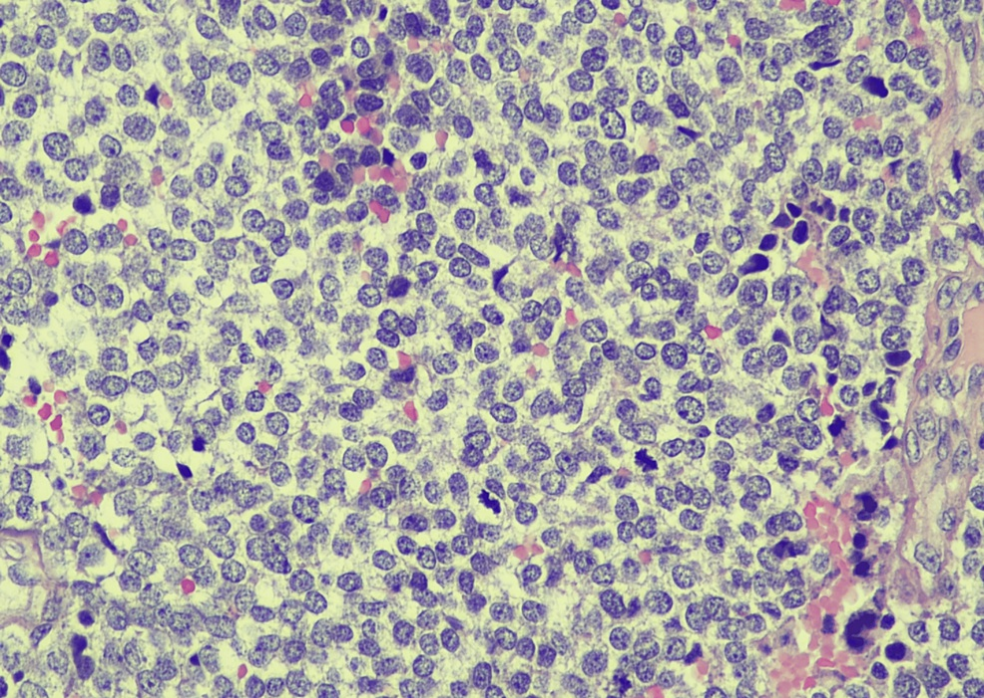
A representative photomicrograph with H&E staining of the initial mass shows a primitive round blue cell sarcoma with fine powdery chromatin and scant amphophilic to somewhat clear cytoplasm (40x magnification). H&E: Hematoxylin and eosin

The mass compressed the ureters bilaterally and required the placement of nephrostomy tubes. Pathology showed a primitive round blue cell sarcoma with high mitotic activity and tumor necrosis (Figure 2). Immunohistochemistry was performed, and genetic testing revealed a characteristic 5’- 3’ EWSR1:FLI gene fusion, and the diagnosis of EwS was irrefutably confirmed. The subsequent PET scan was negative.

**Figure 3:**
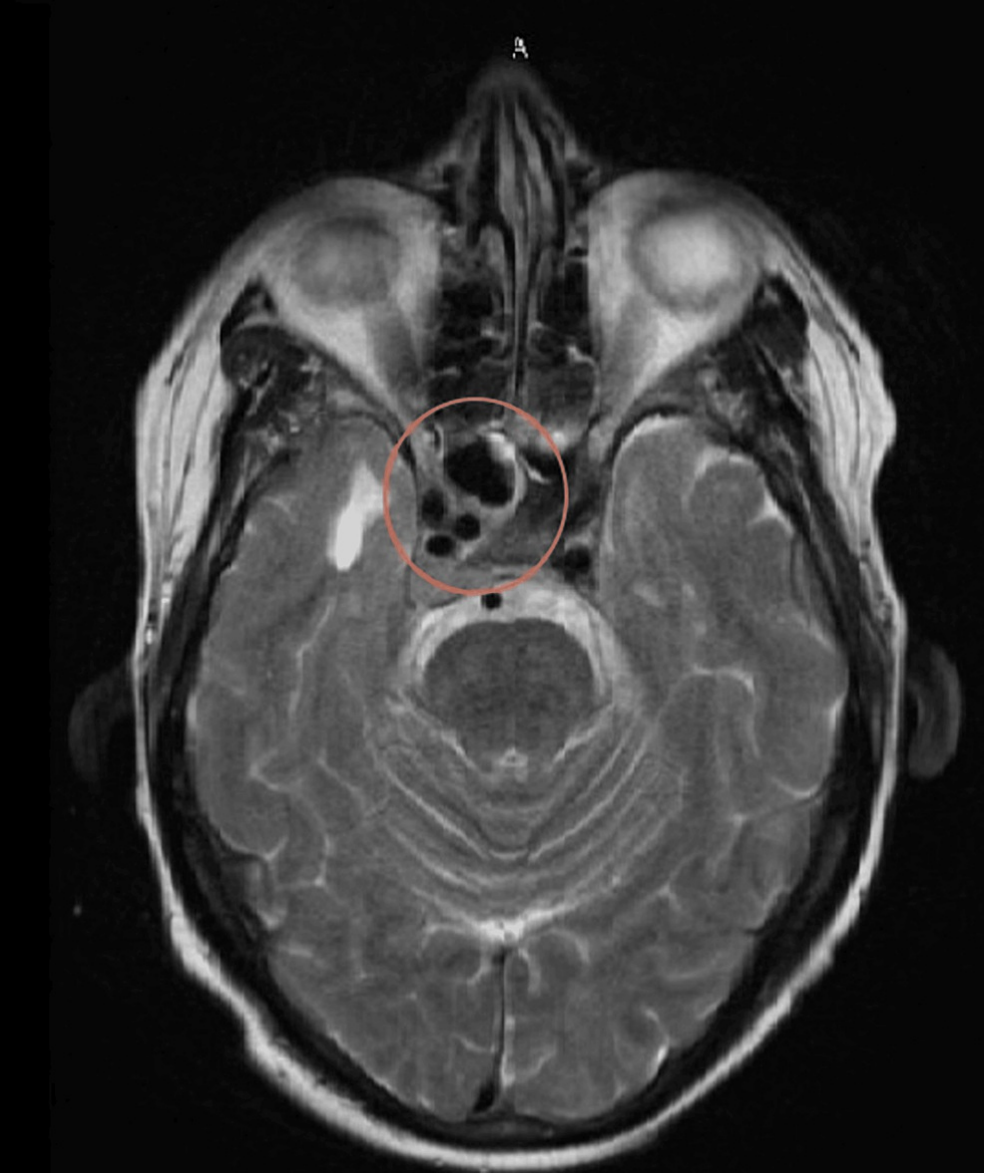
MRI of the brain showing enhancing metastasis along the right cavernous sinus region

The initial plan was to reduce the size of the mass with brachytherapy, followed by a multiagent chemotherapy regimen consisting of alternating cycles of vincristine (2 mg/m2), doxorubicin (75 mg/m2), cyclophosphamide (1200 mg/m2), followed by ifosfamide (9 mg/m2), and etoposide (500 mg/m2). The patient was started on pelvic brachytherapy with 900 cGy delivered over three fractions, including the area of the tumor with a wide margin, and chemotherapy with the aforementioned regimen was initiated. Notable shrinkage in mass was seen on an MRI after the second cycle (shown in Figure 1), but treatment was complicated by toxicity from chemotherapy in the form of weakness, nausea, vomiting, and diarrhea, and eventually a loss of follow-up for four months. The patient then presented to the ER with double vision and headaches, and an MRI showed evidence of metastasis along the right cavernous sinus (Figure 3). Additionally, the patient also had bone pain, and a bone marrow biopsy showed neoplastic cells. Fluorescence in situ hybridization (FISH) analysis was positive for EWSR1 (22q12) rearrangements, indicating the presence of EwS consistent with bone marrow spread and metastases.

## Discussion

In a recent review of the literature on the few cases of vaginal EwS by Machado et al., it was noted that almost all patients presented with a painless vaginal mass [2]. The case presented here came with pelvic pain and intermittent bleeding requiring analgesics and blood transfusions, likely due to the size of the mass. Imaging is important to characterize the mass. Extraskeletal tumors show hypervascular soft tissue mass with areas of necrosis and hemorrhage. These tumors may spread to nearby bones but typically spare the bone marrow [7]. This was not the case with our patient. Approximately 25% of cases have calcifications. A CT scan usually shows the presence of a dense pelvic mass, which is useful to assess the extent of the tumor and will reveal locoregional metastases if present. Whole-body PET/CTs play an important role in evaluating therapeutic responses.

The most common histological features of ES are those consistent with tumors of neuroectodermal origin: small, round cells with a high nucleus-to-cytoplasm ratio and finely dispersed chromatin. Rosettes have also been described. The cells are also often necrotic [8]. When it comes to immunohistochemistry, CD-99 and FLI-1 are the most commonly used markers, but their accuracy has been controversial, and more recently, NKX2.2, with a sensitivity and specificity of around 90%, is a useful alternative [9].

Most literature recommends the management of extraskeletal EwS/primitive neuroectodermal tumors (PNET) with wide excision followed by chemotherapy or radiation, as opposed to skeletal masses, in which surgery is delayed. These tumors are known to be sensitive to radiation [10]. However, for larger, more centrally located masses that are close to large vessels and organs where wide excision may prove difficult (as in this case), it is acceptable to initiate radiotherapy first [11]. When chemotherapy is used, guidelines currently recommend alternating cycles of vincristine, doxorubicin, and cyclophosphamide, along with ifosfamide and etoposide (VDC/IE). Most studies show that this regimen improves event-free survival as well as overall survival [12]. It is important to note that these trials have mostly been conducted in children and individuals under the age of 50 and in patients with skeletal EwS. These tumors, however, have the same mesenchymal origin and should respond to the same treatment. The patient presented here was not a surgical candidate, given the large size of the mass, the largest that we have seen so far across the literature in the female genital tract [13].

## Conclusions

Given the rarity of this type of cancer, further reporting of cases must be encouraged to characterize the clinical, immunohistological, and radiographic features of EwS as well as its response to therapy. Larger masses may be approached with radiation first, as opposed to conventional surgical first-line management. There are presently no clear guidelines for management, but a multi-disciplinary approach is essential. Additionally, molecular analysis may provide further insights into the unconventional location of this cancer.
